# Primary chemotherapy with gemcitabine, epirubicin and taxol (GET) in operable breast cancer: a phase II study

**DOI:** 10.1038/sj.bjc.6602723

**Published:** 2005-07-26

**Authors:** P F Conte, S Donati, A Gennari, V Guarneri, C Orlandini, M Rondini, M Roncella, L Marini, P Collecchi, P Viacava, A G Naccarato, R Degli Esposti, S Bonardi, A Bottini, S Saracchini, S Tumolo, G Gullo, A Santoro, L Crino

**Affiliations:** 1 Department of Medical Oncology and Hematology, University of Modena and Reggio Emilia, Modena, Italy; 2 Division of Oncology, S Chiara University Hospital, Pisa, Italy; 3 Division of Surgery, S Chiara University Hospital, Pisa, Italy; 4 Eli Lilly Italy, Medical Department, Firenze, Italy; 5 Division of Pathology, S Chiara University Hospital, Pisa, Italy; 6 Division of Medical Oncology, Bellaria Hospital, Bologna, Italy; 7 Breast Unit, Istituti Ospitalieri, Cremona, Italy; 8 Division of Oncology, S Maria Angeli Hospital, Pordenone, Italy; 9 Division of Oncology, Istituto Clinico Humanitas, Rozzano, Italy

**Keywords:** gemcitabine, epirubicin, taxol, breast cancer, primary chemotherapy, pathological complete response

## Abstract

This trial was conducted to assess the activity and tolerability of the gemcitabine, epirubicin, taxol triplet combination in patients with operable breast cancer. After core biopsy, 43 women with stage II–IIIA breast cancer were treated with gemcitabine 1000 mg m^−2^ over 30 min on days 1 and 4, epirubicin 90 mg m^−2^ as an intravenous bolus on day 1, and taxol 175 mg m^−2^ as a 3-h infusion on day 1, every 21 days for four cycles. The primary end point was the percentage of pathological complete responses (pCR) in the breast; secondary end points were tolerability, clinical response rates, overall and progression-free survival, tumour biomarkers before and after primary chemotherapy (PCT). All patients were included in safety and survival analyses; 41 eligible patients were evaluated for response. The overall clinical response rate was 87.8% (95% CI 77.8–97.8), with 26.8% complete responses (95% CI 13.3–40.3). A pCR in the breast was observed in six patients (14.6%; 95% CI 3.8–25.4); 15 patients (36.6%; 95% CI 21.9–51.3) had negative axillary lymph nodes. Grade 4 neutropenia was observed in 67.4% of the patients; febrile neutropenia occurred in 1.9% of cycles (granulocyte colony-stimulating factor was used in 3.2% of the cycles to shorten the duration of neutropenia). A statistically significant difference between Mib-1 at baseline (⩾20% in 71.4% of the patients) and at definitive surgery (28.6%, *P*<0.05) was observed. The gemcitabine, epirubicin, taxol regimen is active and well tolerated as PCT for operable breast cancer. This combination allows the administration of full doses of active agents with a low incidence of febrile neutropenia.

Primary chemotherapy (PCT) was initially employed to treat locally advanced and/or inflammatory breast carcinomas. An objective response was observed in more than 50% of the patients, radical surgery was feasible in most of the cases, and prolonged disease-free and overall survival were reported (Hortobagyi and Buzdar, 1997; Ueno *et al*, 1997; Baldini *et al*, 2003). More recently, PCT has been used in patients with large primary tumours to avoid radical mastectomy. Again, a significant proportion of patients experienced an objective response and could be treated with conservative surgery (Bonadonna *et al*, 1990, 1998; Smith *et al*, 1995; Mauriac *et al*, 1999). Interestingly, in about 10% of cases, tumour shrinkage was so massive that no viable tumour cells were found at definitive pathology; the achievement of a pathological complete response (pCR) was associated with a prolonged disease-free and overall survival (Fisher *et al*, 1998; Kuerer *et al*, 1999; Pierga *et al*, 2000). So far, randomised clinical trials have failed to show a survival advantage for PCT *vs* postoperative treatment; however, other advantages such as the increased rate of breast conservative surgery and the prognostic value of pCRs have been confirmed (Scholl *et al*, 1994; Fisher *et al*, 1997; Kuerer *et al*, 1999; Pierga *et al*, 2000; van der Hage *et al*, 2001; Mauri *et al*, 2005).

Primary chemotherapy is now considered the standard treatment for locally advanced and inflammatory breast carcinoma, a reasonable approach for operable breast cancer with unfavourable breast/tumour ratio and an acceptable alternative for all early breast cancer patients who are candidates for adjuvant chemotherapy.

Apart from these established roles in routine practice, PCT represents an interesting research tool which allows investigators to: (1) test new regimens with a validated short-term end point (pCR); (2) design tailored treatments based on the observed response; (3) identify tumour biomarkers with prognostic and/or predictive value.

We have recently shown that the combination of gemcitabine plus epirubicin and taxol (GET) is feasible and extremely active in metastatic breast cancer (Conte *et al*, 2001; Cappuzzo *et al*, 2004). Based on the high overall and complete response rates obtained with this regimen, we have designed a phase II trial to evaluate the activity of GET as PCT in operable breast cancer. An ancillary study was the evaluation of the tumour biological profile before and after chemotherapy. In particular, markers of proliferation have already been validated as valuable predictors of response to both PCT and endocrine therapy, and were therefore included in our study (Esteva and Hortobagyi, 2004; Burcombe *et al*, 2005; Dowsett *et al*, 2005).

Preliminary results from this trial have been previously reported (Conte *et al*, 2003).

## PATIENTS AND METHODS

Patients with histologic diagnosis of breast cancer who met the following criteria were eligible: stage II–IIIa (tumour size ⩾2 cm) determined by physical examination and mammography; adequate bone marrow reserve (white blood cell count ⩾4.0 × 10^9^ l^−1^, platelets ⩾100 × 10^9^ l^−1^, haemoglobin ⩾100 g l^−1^); adequate renal and hepatic function (creatinine <1.5 mg dl^−1^, alanine transaminase or aspartate transaminase <1.5 upper limit of normal (ULN)); normal cardiac function determined by electrocardiogram and left ventricular ejection fraction (L-VEF); World Health Organization (WHO) performance status ⩽2; aged 18–70 years; compliance and geographic proximity; childbearing potential terminated or attenuated by use of an approved contraceptive method; written informed consent. The noneligibility criteria were: locally advanced disease (stage IIIB) or inflammatory breast carcinoma; multifocal breast carcinoma; active infection; calcium above the ULN; presence of distant metastases; other serious medical illness (including history of congestive heart failure, myocardial infarction, symptomatic cardiac arrhythmias). Pre-study staging included physical examination, mammography and/or ultrasonography of the breast, chest radiography, bone scan, liver ultrasonography, echocardiography and haematological blood tests.

### Treatment plan

After core biopsy, patients were treated according to the following schedule: gemcitabine 1000 mg m^−2^ over 30 min on days 1 and 4, epirubicin 90 mg m^−2^ as an intravenous (i.v.) bolus on day 1 and taxol 175 mg m^−2^ as a 3-h infusion on day 1, every 21 days for four cycles. In order to prevent severe hypersensitivity reactions, patients received a premedication with dexamethasone 20 mg i.m., orphenadrine 50 mg i.m. and cimetidine 300 mg i.v.

Complete blood count was determined on day one of each cycle and then twice a week. Treatment was permitted if white blood cell count was ⩾3.0 × 10^9^ l^−1^, absolute neutrophil count was ⩾1.5 × 10^9^ l^−1^ and platelet count was ⩾100 × 10^9^ l^−1^; if these values were not reached, treatment was delayed until recovery. All drugs were reduced by 25% in case of febrile neutropenia requiring hospitalisation and/or i.v. antibiotics, grade 4 thrombocytopenia lasting more than 3 days and/or associated with bleeding, or grade 4 neutropenia lasting more than 7 days. In case of grade 3 nonhaematological toxicities (except nausea/vomiting and alopecia), all drugs were reduced by 25%; all drugs were reduced by 50% for grade 4 toxicity. In the case of grade 1 neurotoxicity, taxol was reduced to 135 mg m^−2^, and in the case of grade 2 neurotoxicity, taxol was discontinued. The protocol permitted use of granulocyte colony-stimulating factor (G-CSF) as prophylaxis after episodes of febrile neutropenia. Patients with hypersensitivity reactions received supportive measures and were treated again with taxol at a slower rate of infusion.

Patients who developed progressive disease were taken off the study.

### Surgery

After four cycles of chemotherapy, breast surgery was performed according to local procedures. Patients received either modified radical mastectomy or lumpectomy with axillary dissection of level I–II axillary lymph nodes.

### Adjuvant therapy

Adjuvant postoperative chemotherapy was left to the discretion of the treating physician; however, two additional courses of chemotherapy were recommended in cases of positive lymph nodes. Premenopausal receptor-positive patients received tamoxifen 20 mg daily for 5 years plus luteinising hormone-releasing hormone inhibitors for 2 years. Postmenopausal receptor-positive patients received tamoxifen 20 mg daily for 5 years.

In cases of breast conservative surgery, postoperative irradiation with a 4–6 MeV linear accelerator or modern cobalt-60 unit was administered. Radiotherapy was started after completing chemotherapy.

### Study analysis

#### Assessment of response

Clinical assessment of tumour and nodal size was performed by physical examination, mammography and/or ultrasonography before starting chemotherapy, and again before surgery.

Clinical response was defined as follows: complete response is the disappearance of all clinically detectable disease; partial response is a ⩾50% decrease in the products of the two largest tumour diameters; stable disease reflects changes in tumour burden that do not indicate a progressive disease or clinical complete or partial response. Progressive disease was defined as at least a 25% increase in the sum of the products of bidimensionally measurable disease or the appearance of new lesions.

Pathological complete response was defined as no histologic evidence of invasive or noninvasive tumour cells in the breast. Toxicity was evaluated according to the common toxicity criteria of the National Cancer Institute. Progression-free and overall survivals were calculated according to Kaplan–Meier curves from day 1 of the first cycle. All registered patients were included in the analysis of toxicities and survival. Eligible patients only were included in the primary efficacy analysis.

#### Biological study

The following biological markers were assayed at baseline and on the surgical specimens: hormone receptor, Mib-1, Scarff–Bloom–Richardson grade and Her-2-neu expression. All the biological markers were measured in a centralised laboratory.

#### End points

The primary end point was the pCR rate. Secondary end points were: toxicities, overall response rate, survival and progression-free survival, and tumour biological profile before and after PCT.

#### Statistical methods

Simon's optimal two-stage design for phase II clinical trial was used to calculate the sample size, with the principal study end point being the pCR rate. The sample size was calculated on the following assumptions: *α* error=0.05, *β* error=0.10; *P*_0_ (clinically uninteresting true response rate) and *P*_1_ (sufficiently promising true response rate) were set at 5 and 20%, respectively. In all, 21 patients had to be enrolled in the first stage: if ⩽1 pCR were observed, the accrual was stopped. In case of ⩾2 pCR, 20 more patients were entered at the second stage. The regimen was considered sufficiently active to deserve further studies if ⩾5 pCRs were seen.

## RESULTS

### Patient characteristics

A total of 44 women with stage II–IIIA disease from five Italian institutions were enrolled into the study; one patient was not included in the full analysis set for refusal. In all, 41 patients were eligible, two patients were not included in the primary efficacy analysis due to the existence of bilateral breast carcinoma. The characteristics of the patients are shown in Table 1. The median age was 48 years (range 26–66 years); the majority of patients had stage II disease (67.4%) and positive hormone receptors (79.1%). Baseline hormonal status was not available in one patient who achieved a pCR. Median tumour size at the baseline was 4 cm (range 2–7 cm). Mib-1 was ⩾20% in 23 (53.5%), <20% in 13 (30.2%) and not known in seven patients. Her-2 status was positive in five (11.6%), negative in 25 (58.1%) and unknown in 13 patients.

### Response to chemotherapy

All eligible patients were evaluable for response. The overall clinical response rate was 87.8% (95% CI 77.8–97.8), with 61% of the patients experiencing a partial response and 26.8% (95% CI 13.3–40.3) a complete response; stable disease was observed in five patients (12.2%).

A pCR was observed in six patients (14.6%) (95% CI 3.8–25.4); 15 patients (36.6%) (95% CI 21.9–51.3) had negative axillary lymph nodes.

The pCR rate was analysed as a function of pretreatment clinical stage and biological characteristics. Two pCRs were observed among five patients with tumours less than 3 cm (40%), three among 25 women with tumour size between 3 and 5 cm (12%), and one in 11 patients with tumours larger than 5 cm (9.1%). The pCR rate was 9.7% (three out of 32) in oestrogen receptor-positive and 25% (two out of eight) in oestrogen receptor-negative tumours, 17.4% (four out of 23) in Mib-1 ⩾20 and 7.7% (one out of 13) in Mib-1 <20% tumours, 20% (one out of five) and 4% (one out of 25) in Her-2-positive and -negative tumours, respectively; none of these differences reached statistical significance.

**Figure 1:**
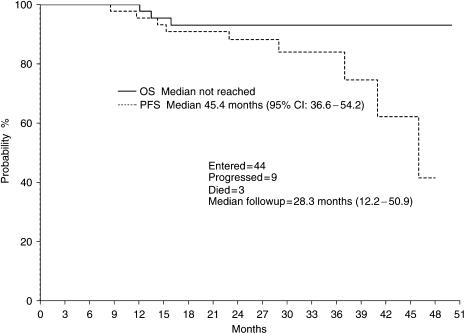
Progression-free survival and overall survival.

At a median followup of 29 months (range 12.2–51), nine patients had developed progressive disease and three patients had died (Figure 1).

### Toxicity

In all, 43 patients were evaluable for toxicity. During the study, 172 cycles of chemotherapy were administered and were evaluable for toxicity.

The main haematological toxicity was neutropenia, with grade 4 episodes observed in 67.4% of the patients. Febrile neutropenia occurred in 1.9% of cycles, while G-CSF was administered in 3.2% of the cycles to shorten the duration of neutropenia. Only one episode of grade 3 anaemia was observed, while thrombocytopenia was mild, with 9.3% of patients experiencing a grade 3 toxicity (Table 2). One red blood cell transfusion was performed, but no platelet transfusion was required. Delays and dose reductions were performed in 10.3 and 9.7% of the cycles, respectively. The administered dose intensity of each individual drug was 95, 98 and 97% for gemcitabine, epirubicin and taxol, respectively.

No episode of grade 4 nonhaematological toxicity occurred. Nausea/vomiting and mucositis grade 3 were observed in 9.3 and 7% of the patients, respectively. Grade 1–2 neuropathy was observed in 18.6% of the patients. Cutaneous toxicity was mild, with 41.9% of the patients experiencing grade 1–2 toxicity (Table 3).

No clinical cardiac toxicity or significant decrease of L-VEF was observed.

### Surgery

All the patients underwent surgery for breast cancer. In all, 19 patients (44%) were treated with lumpectomy and axillary node dissection, and 24 patients (56%) received modified radical mastectomy.

### Biological study

The expression of hormonal receptor status and nuclear grading were not modified by chemotherapy. A statistically significant difference between Mib-1 at baseline and at definitive surgery was observed: Mib-1 ⩾20% in 71.4% of the patients at baseline and in 28.6% at surgery (*P*<0.05). Her-2-neu expression was also modified by chemotherapy even if the difference was not significant: it was positive in 21% of the patients at baseline and in 10.5% at surgery. Biological data are summarised in Table 4.

## DISCUSSION

International recommendations have been developed to support the use of PCT in routine practice (Kaufmann *et al*, 2003); however, this approach still represents an interesting research tool that allows a reliable intermediate end point such as pCR to be measured, treatments to be designed and tailored on response, and tumour biomarkers with potential prognostic or predictive value to be investigated. Our study was designed to assess the efficacy and tolerability of the GET regimen as PCT in operable breast carcinoma. This combination was initially tested in metastatic breast cancer patients, showing an interesting activity with response rates ranging from 62 to 92%, with 10–31% CR (Conte *et al*, 2001; Zielinski *et al*, 2003; Cappuzzo *et al*, 2004). This regimen was found to be well tolerated. The majority of the patients experienced a grade 4 neutropenia; however, the incidence of febrile neutropenia was very low (5–12%) and growth factors were rarely used.

This trial confirms that the GET regimen can be safely administered at full doses with a few patients requiring dose adjustments. The incidence of grade 4 neutropenia was high (67.4% of the patients); however, it was short lasting, rarely requiring G-CSF administration (3.2% of cycles), and resulted in very few episodes of febrile neutropenia (1.9% of cycles), significantly lower than that observed in an adjuvant trial with a three-drug regimen including docetaxel, doxorubicin and cyclophosphamide (Martin *et al*, 2003). The reduced myelotoxicity of paclitaxel *vs* docetaxel, epirubicin *vs* doxorubicin, gemcitabine *vs* cyclophosphamide, and the lack of negative pharmacokinetic interference explain the good tolerability of the GET regimen (Fogli *et al*, 2002). The more favourable patient characteristics (younger median age, excellent performance status) and the shorter treatment duration (four courses in the present trial, six to eight courses in the GET trials in advanced breast cancer) can explain the extremely low incidence of febrile neutropenia observed in the present trial in comparison to the other trials with the GET regimen (Conte *et al*, 2001; Zielinski *et al*, 2003; Cappuzzo *et al*, 2004).

The primary aim of our study was to evaluate the activity of the GET combination. The overall response rate was 87.8%, with 26.8% clinical complete remissions and 14.6% pCRs.

Conservative surgery was not an end point of the study and no guideline for conservative surgery was included in the protocol; this reason and the fact that the patients were treated in five different hospitals may explain the relatively low percentage of conservative surgery (44%). The high activity of this triplet regimen has been confirmed by Hamm *et al* (2003) in locally advanced breast cancer, and by Schneeweiss *et al* (2004) in operable breast cancer. In this last trial, paclitaxel was substituted with docetaxel (75 mg m^−2^), the dose of gemcitabine was reduced to 800 mg m^−2^ on days 1 and 8, epirubicin was administered at 90 mg m^−2^ and filgrastim support was required.

Comparable pCR rates have been obtained with other regimens containing taxanes, anthracyclines or antimetabolites (Gradishar, 1997; Ganem *et al*, 2003; Dieras *et al*, 2004; Lebowitz *et al*, 2004; Smith *et al*, 2004). Differences in patient selection, tumour size and characteristics, treatment duration and pathological classification of response make any comparison impossible. It is, however, of interest that the GET regimen allows the concurrent administration of full doses of active drugs, thus producing results comparable to those obtained with more prolonged sequential schedules (Gianni *et al*, 2002; Smith *et al*, 2002; Bear *et al*, 2003; von Minckwitz *et al*, 2003).

Primary chemotherapy provides the ideal setting to investigate prognostic and predictive factors; apart from pCR, the most powerful predictor of long-term outcome is the status of axillary lymph nodes. In our study, 36.6% of the patients had negative axillary lymph nodes at surgery. However, the nodal status was not pathologically documented at study entry, and it is therefore impossible to measure the effect of treatment on this parameter. Many predictive factors of response have been studied, and several authors have reported that hormone receptor negativity, high histological grade and high Ki-67 levels correlate with pCR, while the predictive value of Her 2 expression, bcl-2 and p53 status is still unclear (Archer *et al*, 1995; Makris *et al*, 1997; Chang *et al*, 1999; Colleoni *et al*, 1999; Ellis *et al*, 2001; Petit *et al*, 2001). In our study the probability of pCR was higher, even if statistically not significant, in the case of hormone receptor-negative tumours (25 *vs* 9.7%), Mib-1 ⩾20% (17.4 *vs* 7.7%) and Her-2 overexpression (20 *vs* 4%).

Availability of tumour tissue before, during and after PCT allows for the possibility of measuring biomarker expression during treatment and, potentially, correlating the observed modifications with outcome. In particular, the tumour-proliferative rate measured as Ki-67, Mib-1 or thymidine labelling index is rapidly and significantly inhibited by hormonal treatment or chemotherapy (Collecchi *et al*, 1998; Colleoni *et al*, 1999; Archer *et al*, 2003). We have previously reported in locally advanced breast cancer studies that inhibition of tumour proliferation after PCT predicts a better outcome (Collecchi *et al*, 1998). In this study, we have shown that Mib-1 level was significantly lower after four courses of GET (Mib-1 ⩾20% in 71.4 and in 21.6% of the patients before and after chemotherapy, respectively, *P*<0.05); however, the limited sample size and the low number of events do not allow this finding to be correlated with patient outcome.

Primary chemotherapy can facilitate the development of treatments tailored on the quality of response (pCR *vs* non-pCR) and biomarker expression and modulation. In this setting, the expression of gene profile measured by DNA microarray offers the opportunity to identify predictive markers of response; this allows us to identify the patients who benefit from the treatment and to spare unnecessary toxicities to those with *de novo* resistant tumours (Buchholz *et al*, 2002; van de Vijver *et al*, 2002; van't Veer *et al*, 2002; Chang *et al*, 2003; Pusztai *et al*, 2003).

In conclusion, we have shown that four courses of preoperative GET are safe and highly active in patients with early-stage breast cancer. This tolerability is worth noting, taking into account that all drugs were administered at nearly full doses, and this regimen can represent an alternative to the sequential administration of myelotoxic drugs. However, in order to exploit all the opportunities offered by preoperative treatments, a coordinated multidisciplinary approach including clinical oncologists, pathologists and molecular biologists is required (Buchholz *et al*, 2003).

## Figures and Tables

**Table 1:** Patient characteristics

Number of patients enrolled	44
Patients eligible	41
Median age (range)	48 (26–66)
Median tumour size, cm (range)	4 (2–7)
	
*Hormonal receptor status, n* (%)	
Positive	34 (79.1)
Negative	8 (18.6)
Unknown	1 (2.3)
	
*Mib-1 status, n* (%)	
Positive (⩾20%)	23 (53.5)
Negative (<20%)	13 (30.2)
Unknown	7 (16.3)
	
*Her 2 status, n* (%)	
Positive	5 (11.6)
Negative	25 (58.1)
Unknown	13 (30.2)

**Table 2:** Haematological toxicity (% of patients)

	**Grade 1**	**Grade 2**	**Grade 3**	**Grade 4**
Anaemia	53.5	27.9	2.3	—
Neutropenia	2.3	9.3	16.3	67.4
Leucopenia	7.0	30.2	44.2	14.0
Thrombocytopenia	34.9	16.3	9.3	—

**Table 3:** Non-haematological toxicity (% of patients)

	**Grade 1**	**Grade 2**	**Grade 3**	**Grade 4**
Nausea/vomiting	39.5	37.2	9.3	—
Mucositis	27.9	9.3	7.0	—
Paresthesias	16.3	2.3	—	—
Cutaneous	27.9	14.0	—	—
Diarrhoea	11.6	0.0	2.3	—
Alopecia	—	4.7	95.3	—

**Table 4:** Modification of tumour phenotype

	**Pretreatment (% of patients)**	**Post-treatment (% of patients)**	***P***
Oestrogen-receptor positive	69.7	73.9	0.90
Grade 3	68.5	72.7	0.96
Mib 1 ⩾20%	71.4	28.6	<0.05
Her-2 neu	21.0	10.5	0.46
